# Distribution of transgene in the rodent choroid plexus after intracerebroventricular injection of adeno-associated virus

**DOI:** 10.1186/s12987-026-00831-4

**Published:** 2026-07-31

**Authors:** John R. Pooley, Ali S. Bienemann, Alison Young, Owen Hollings, Jiahui Wu, Eddie Mayo, Colin J. Chu, Will G. B. Singleton

**Affiliations:** 1 Translational Health Sciences, Bristol Medical School, University of Bristol, Bristol, BS8 1TD UK; 2 Academic Unit of Ophthalmology, Bristol Medical School, University of Bristol, Bristol, BS8 1TD UK; 3 Translational Biomedical Research Centre, Bristol Medical School, University of Bristol, Bristol, BS40 5DU UK; 4 Institute of Ophthalmology, University College London, London, EC1V 9EL UK; 5 National Institute for Health Research Biomedical Research Centre, Moorfields Eye Hospital, London, EC1V 2PD UK; 6 Department of Paediatric Neurosurgery, Bristol Royal Hospital for Children, University Hospitals Bristol and Weston NHS Foundation Trust, Bristol, BS2 8BJ UK

**Keywords:** Choroid plexus, Gene therapy, Intracerebroventricular, AAV, CRISPR, Aquaporin, Distribution, ShH10, SaCas9, Knockdown

## Abstract

Using adeno-associated virus to transfer genetic information to the choroid plexus has emerged as a promising route for long-term gene therapy in the brain for a variety of conditions. Overexpression of proteins has proved effective in small animal models but few attempts have been made to translate this technology to clinic, suppress the activity of a protein of interest, or further expand the limited capsid serotypes known to have choroid plexus tropism. We utilise transfer of green fluorescent protein to show choroid plexus epithelium tropism for novel AAV6 derived capsid ShH10^Y445F^ in mouse, rat and porcine tissue explant cultures. In vivo tropism is shown in the mouse following stereotactic intracerebroventricular injection. We examined the distribution of viral transduction across the choroid plexus in all four ventricles following a single unilateral intracerebroventricular injection using both green fluorescent protein as a transgene, but also the CRISPR/Cas9 system to deliver permanent knockdown of apical water channel aquaporin-1. Quantitative immunofluorescence and SURVEYOR assay were used to statistically assess the magnitude and extent of choroid plexus knockdown across the ventricular system. We conclude that serotype ShH10^Y445F^ targets choroid plexus epithelium in mouse, rat and pig; and when carrying the CRISPR/Cas9 system can reduce target protein expression in these cells. Transduced choroid plexus epithelial cells distribute unevenly with a bias toward the lateral ventricle on the injected side and a preferential infection of choroid plexus in the lateral over the third and fourth ventricles. Overcoming irregular distribution represents a challenge for clinical translation of this technology where clinical efficacy may require manipulation of the entire choroid plexus.

**Supplementary Information:**

The online version contains supplementary material available at 10.1186/s12987-026-00831-4.

## Introduction

Application of adeno-associated virus (AAV) for central nervous system (CNS) gene therapy offers several advantages; including minimal immune response, a strong safety profile supported by existing approved treatments, and sustained long-term transgene expression suitable for lifelong conditions [1]. As a non-integrating vector, AAV persists as episomal DNA, reducing the risk of oncogenesis [2]. Its relatively small particle size (22–25 nm) [3], combined with a wide and expanding range of serotypes with diverse tropisms enables efficient and widespread distribution. AAV is relatively straightforward to manufacture and can be delivered using simple, potentially single-injection approaches with standard equipment, while direct administration into the CNS helps minimise systemic toxicity and off-target effects [4].

Infection of antigen presenting cells in brain parenchyma, particularly astrocytes and microglia, has led to undesirable negative immune consequences [5, 6]. Bajocchi and colleagues proposed an alternative approach for delivering AAV into the brain [7], where injection into cerebrospinal fluid (CSF) via the lateral ventricle caused infection and transgene expression in the ependyma, the epithelial cells lining CSF pathways. Later the choroid plexus epithelium (ChPE) was added as a further target for therapeutic transgene production [8–10].

The choroid plexus (ChP) is a specialised epithelial tissue within all four ventricles of the brain. A single layer of polarised epithelium connected by apical tight junctions forms the barrier with the CSF compartment and restricts the passage of molecules [10, 11]. The ChPE is post-mitotic so turns over very slowly [10, 12–14], while actively producing CSF through active ion transport driven by Na/K ATPase [15, 16] and water passage through an ion cotransporter [17, 18]. The water channel aquaporin-1 (AQP1) is expressed at high levels on the apical surface of the ChPE and contributes to the water permeability of this membrane [19]. Multiple authors have suggested a potential for AQP1 inhibition as a novel therapeutic strategy to treat CSF hypersecretion subsequent to intra-ventricular haemorrhage that may contribute to the development of post-haemorrhagic hydrocephalus [20–22], but its role is controversial [17]. Nonetheless, reducing the activity of this protein with conventional pharmaceuticals has been challenging [20, 23], making AQP1 an interesting target for genetic manipulation.

Gene therapy proposals targeting the ChP and/or ependyma for the purposes of therapeutic benefit have been developed in mouse models. Efficacy has so far been demonstrated across a range of transgenes in models of multiple sclerosis [11, 24], Alzheimer’s disease [25], congenital obstructive and post-haemorrhagic hydrocephalus [26, 27]. These studies sought to overexpress a therapeutic protein. In contrast, little attention has been afforded to the possibility of reducing protein expression by selective gene knockdown. Targeting the ChP via ICV injection is a workable adaptation of this approach as demonstrated in a mouse model of multiple sclerosis wherein the adenosine A2A receptor was knocked down to therapeutic benefit [11].

Transduction of ependyma and ChP by AAV varies with serotype but also by route of administration, dose, and post injection time frame in mice [28, 29], with consensus not yet reached on which serotypes are best for particular cell types [30]. Serotypes AAV1, AAV2/1, AAV2/2, AAV4, AAV2/4, AAV8, AAV2/8, AAV2/5, AAV2/9 and AAV-rh10 have been shown to transduce ChP with varied efficiency and selectivity [9, 12, 28, 31–39]. Many also maintain tropism for other CNS cell types including neurons and glia, particularly in the cerebellum and cortex. AAV2/5, AAV2/8 and AAV2/1 have emerged from mouse studies as favoured serotypes for ChP transduction [33].

AAV2/6 and related AAV2/1 possess similar transduction patterns with sparse but widespread transgene expression across the cortex, ChP and ependyma in cats [28]. AAV2/6 also infects retinal pigment epithelium following subretinal injection [40] and can target airway and oviduct epithelia [41, 42]. In the present investigation we report ChPE tropism for a novel AAV6 capsid variant that does not occur in nature and may therefore be less likely to undergo neutralisation by preexisting antibodies in humans. The ShH10 variant of AAV6, produced from a capsid shuffle directed evolution approach [43] transduces retinal pigment epithelium with similar strength as AAV2/5 [44], while we and others have shown capacity to infect ciliary body epithelium upon intravitreal injection [45, 46]. Rational design approaches advocate the inclusion of a Y445F change to enhance transduction efficiency, deliver greater tropism for epithelial cells and boost resistance to neutralisation by preexisting antibodies [47, 48].

In this study, we show serotype AAV2/ShH10^Y445F^, which carries the AAV2 genome with an ShH10^Y445F^ capsid protein, infects ChPE in mice, rats and pigs; demonstrating broad cross-species application. In the mouse brain this serotype demonstrated strong transgene overexpression with good selectively to the ChPE and little or no ependymal co-infection unlike AAV2/1 [9, 32]. A single unilateral ICV injection site spread AAV throughout the ventricular system including into the contralateral ventricle, but transgene expression occurred along a rostro-caudal gradient with areas closer to the injection site being better targeted than those further away. We show clustered regularly interspaced short palindromic repeats (CRISPR)/Cas9-mediated AQP1 protein reduction in the ChPE is possible, but efficacy also follows a concentration gradient after a single ICV injection.

## Materials and methods

### AAV cloning and manufacture

Derivation of AAV containing SaCas9 and guide RNA sequences 1B and 1E for exon 1 targeting of the mouse Aqp1 gene has already been described [45]. Plasmid pD10-CMV-EGFP used to generate AAV for enhanced green fluorescent protein (EGFP) transgene delivery and the helper plasmid HGTI have previously been described [49]. Plasmid shh10 was a gift from John Flannery and David Schaffer (Addgene plasmid #64867) and carried the ShH10 cap gene [43], with the Y445F mutation introduced by site directed mutagenesis. To produce a non-targeting control (NTC) variant of the SaCas9 plasmids, guide sequences were designed to EGFP then checked against the mouse (GRCm39/mm39, GRCm38/mm10, NCBI37mm9), rat (mRatBN7.2/rn7, RGSC6.0/rn6, RGSC5.0/rn5) and human genomes (GRCh38/hg38, GRCh37/hg19, NCBI36/hg18) with the blat tool in the UCSC Genome Browser [50] to ensure no similar matches. A verified non-targeting guide sequence was cloned into plasmid pX601-AAV-CMV::NLS-SaCas9-NLS-3xHA-bGHpA; U6::BsaI-sgRNA that was a gift from Feng Zhang (Addgene plasmid #61591) [51] using a golden gate protocol. BsaI-HF (New England Biolabs, NEB, Ipswich, UK) cut the vector before insertion of an annealed pair of oligo sequences coding the designated NTC guide (forward sequence 5’-CACCGAAGGGCATCGACTTCAAGGA-3’ and reverse sequence 5’-AAACTCCTTGAAGTCGATGCCCTTC-3’ (Merck)). DNA sequences were joined with T7 DNA ligase (Enzymatics, Beverly, MA) and residual linear DNA removed with PlasmidSafe exonuclease (Thermo Fisher, Loughborough, UK). For pAAV-CMV-SaCas9-U6del an inert part of pcDNA3 was cloned between the KpnI and EagI sites of Addgene plasmid 61,591 above with annealed oligos (forward 5’-CATTGGTACCCACAGAATCAGGGGATAACGCAGGAAAGAACCGGCCGTAAG-3’, reverse 5’-CTTACGGCCGGTTCTTTCCTGCGTTATCCCCTGATTCTGTGGGTACCAATG-3’). This removed the U6 promoter, the scaffolding and guide RNA sequences preventing the SaCas9 from being targeted to the genome.

Plasmids were expanded in E-coli and purified by endotoxin-free megaprep (Qiagen, Dusseldorf, Germany) before triple transfection in the ratio 3:1:1 (HGTI: shh10^Y445F^:transgene) into Lenti-X 293T cells (Clontech, Mountain View, CA) grown in DMEM (#41966, Merck) supplemented with 10% fetal calf serum, 2 mM L-glutamine, 100 units/ml penicillin and 100 µg/ml streptomycin. Polyethylenimine hydrochloride MAX MW 40,000 reagent (PEI, Park Scientific, Northampton, UK) was used to transfect 15 cm plates seeded 24 h previously with 25 million cells. Each 15 cm plate received 37.5 µg total plasmid prepared with PEI in plain DMEM media with no supplements and 20–40 plates were pooled per production run. The following day media on the plates was replaced with 15 ml DMEM fresh media as described above but containing no FCS.

AAV from transfected plates was harvested 72 h after transfection by scraping cells into media and recovering the cell pellet by centrifugation. Media was filtered (0.22 μm pore size) and AAV within it precipitated with polyethylene glycol 8000 (PEG, Merck) prepared in 5 M NaCl and centrifugation (2500 xg, 30 min, 4 °C) after co-incubation on ice for 2 h. Cell pellets were washed and resuspended in TD buffer [140.31 mM NaCl, 24.77 mM Tris base, 4.96 mM KCl, 1.79 mM MgCl_2_, 0.689 mM K_2_HPO_4_] and lysed by 5 cycles of freeze-thaw then addition of sodium deoxycholate. Unpackaged viral DNA and contaminating genomic DNA (gDNA) was removed with benzonase (Merck) digestion at 37 °C for 30 min. Cell debris was pelleted by centrifugation (4000 xg, 30 min, 18 °C) and crude lysate filtered through a 0.45 μm syringe filter (Thermo Fisher). Purification of AAV from lysate was performed using iodixanol (Merck) density gradient ultracentrifugation and the pure fraction washed and concentrated into sterile 1X PBS pH7.4 using centrifugal filter units (Amicon, Harrogate, UK). AAV preparations were titred using qPCR against a standard curve prepared from a double stranded DNA amplicon with Taqman Universal Master Mix II with UNG (Thermo Fisher), unlabelled primers and a FAM-TAMRA labelled probe (Merck). For larger experiments commercial AAV manufacture from our transgene plasmids was provided by Vector Biolabs (Malvern, PA, USA) and was re-titred using qPCR prior to injection.

### Animals and treatments

Adult (20 weeks) female C57BL/6J mice were obtained from Charles River Laboratories (Oxford, UK). Animals were housed with enrichment under a 12:12 h light: dark cycle with lights on at 07:00 and food and water ad libitum. Animal work was performed in accordance with the amended Animals (Scientific Procedures) Act, 1986, and EU Directive 2010/63/EU under project licence PPL P6AAA8252 granted by the UK Home Office, or where procedures were not required, under University Investigation Number UB-22-084. All studies were approved by the Animal Welfare and Ethics Review Body at the University of Bristol. Animals were humanely killed using schedule 1 methods or intraperitoneally injected with 2,105 mg/kg (mice) Euthatal (Duggan Veterinary Supplies Ltd, Coventry, UK) for deep non-recovery anaesthesia and transcardially perfused with 2% formaldehyde in 1X PBS (Thermo Fisher).

Mice were anaesthetised with isoflurane inhalant in an induction chamber then transferred to a rat stereotactic frame (David Kopf Scientific Instruments, Tujunga, CA) adapted for mice by use of a mouse adapter block (World Precision Instruments, Hitchin, UK). An adapted face mask attached to the bite bar continued to supply isoflurane during the procedure, while body temperature was maintained with an Animal Temperature Controller (World Precision Instruments). Fur was shaved from the scalp and viscotears eye gel (Bausch+Lomb, Kingston upon Thames, UK) applied to maintain eye hydration, before draping and preparation of the surgical area with 2% chlorohexidine (Ecolab, Andover, UK). A midline incision was made to expose bregma and lambda and soft tissues anaesthetised with topical lidocaine (20 mg/ml, Hameln Pharmaceuticals Ltd, Gloucester, UK). A 1 mm diameter burr hole was drilled at the injection site on the left side at stereotactic coordinates ML = − 1, AP = 0 relative to bregma. Delivery of AAV or PBS vehicle was performed into the lateral ventricle at 2 mm depth with a 0.5 µl/min infusion rate using a 10 µl Hamilton microsyringe fitted with a blunt-ended, 32-gauge needle (both Thermo Fisher). Following infusion of 10 µl volume the needle remained in situ for 5 min to allow settling before being slowly withdrawn. Skin was closed with sutures and tissue adhesive and the wound dressed with veterinary wound powder (Battles, Lincoln, UK). Mice were further given 5% glucose-saline into the flanks to replace fluids and 4.286 µg/kg buprenorphine (Ceva Animal Health Ltd, High Wycombe, UK) intramuscularly for pain relieve before isoflurane was withdrawn and the animal recovered.

### Primary tissue culture

Primary culture of tissue explants mirrored previously published protocols [52, 53]. ChP was dissected from the lateral and third ventricle of euthanised mice (as above) and rats (Sprague Dawley, 150–175 g, Envigo, Bicester, UK) and rinsed briefly in sterile DPBS. Euthanised pig ChP was obtained from the Translational Biomedical Research Centre at the University of Bristol through the internal Tissue Share Network and stored in ice cold artificial CSF during transport, before dissection into smaller pieces and rinsing in sterile DPBS. Tissue pieces were placed into 6-well culture plates with bases coated with 20 µg/ml laminin (Merck, Gillingham, UK). Culture media comprised of DMEM/HAM’s F12 base medium (#11320074, Thermo Fisher) supplemented with 10% heat-inactivated fetal calf serum (Thermo Fisher), 4 mM additional L-glutamine (Thermo Fisher), 100 units/ml penicillin, 100 µg/ml streptomycin (Thermo Fisher), 200 ng/ml hydrocortisone, 20 µM cytosine arabinofuranoside, and 5 ng/ml sodium selenite (all Merck). Mouse epidermal growth factor (EGF; Bio-Techne, Abingdon, UK), rat EGF (Peprotech, Altringham, UK), or porcine EGF (Generon, Slough, UK) was also added at 10 ng/ml as appropriate to the species. Where appropriate, AAV or vehicle (1X PBS, pH 7.4) was added to cultures on day 0 at 3.189 × 10^10^ gc/ml media. Media was replaced on day 2. On day 4 media was replaced with culture media absent hydrocortisone, cytosine arabinofurandoside, sodium selenite and EGF. On day 7 and 9 media was replaced with serum free growth media (DMEM/HAM’s F12 supplemented with L-glutamine, penicillin and streptomycin as above).

Imaging of living explant cultures was performed at intervals using an inverted EVOS FL Auto Imaging System (Thermo Fisher). On day 10 explant tissue was fixed with a 1:1 mixture of serum free media and 8% formaldehyde in PBS for 2 h at room temperature under gentle agitation. Tissue pieces were rinsed in 1X PBS, cryoprotected at 4 °C overnight with 30% sucrose in PBS, then embedded in Optical Cutting Temperature (OCT) compound (Thermo Fisher) on dry ice and stored at -80 °C until sectioning.

### Fluorescent immunocytochemistry

Fixed brains were dissected from perfused mice and further postfixed in 4% formaldehyde in PBS at 4 °C overnight, washed (3 × 5 min in 1X PBS), then cryoprotected in 30% sucrose in PBS (4 °C until brains sank). Brains were frozen on foil over dry ice and stored at -80 °C until required, then mounted in OCT compound for cutting on a cryostat (Leica Microsystems, Milton Keynes, UK). All tissue sections were cut at 14 μm, thaw mounted onto Superfrost Plus microscope slides (New Erie Scientific LLC, Portsmouth, UK), dried for 10 min at room temperature and then stored at -20 °C. Mounted sections were thawed and air dried fully to allow full adhesion of the tissue section to the slide, then outlined with an ImmEdge hydrophobic marker (Vector Biolabs, Malvern, PA, USA). Adherent cell cultures were fixed in a 1:1 mix of growth media and 8% formaldehyde (Thermo Fisher) for 15 min at room temperature, then washed three times in 1X PBS before proceeding directly to immunocytochemistry.

Tissue slides for native EGFP visualisation were rehydrated 10 min at room temperature with 1X PBS containing 1 µg/ml DAPI (Merck) to stain nuclei. After washing 3 × 10 min in 1X PBS slides were coverslipped with borosilicate glass (thickness No1, VWR, Lutterworth, UK) using aqueous antifade fluorescent mounting medium (Abcam, Cambridge, UK). Imaging was performed initially on an inverted EVOS FL Auto Imaging System.

EGFP signal was recovered on over-fixed mouse explant tissue sections prior to blocking using antigen retrieval with hot 10 mM citrate buffer at pH 6.0. Both cultures and slides were blocked for 2 h at room temperature with blocking solution [1X PBS, 2% BSA, 0.3% Triton X-100]. Samples were washed 2 × 5 min in 1X PBS then primary antibody solution [1X PBS, 0.3% Triton X-100] was added overnight at 4 °C containing primary antibody anti-Aqp1 (EPR11588(B), ab168387, 1:400) or anti-HA tag (ab9110, 1:100, both Abcam). For some slides primary antibody was substituted for a non-immune rabbit IgG as a control (ab172730, Abcam).

After primary incubation samples were washed 3 × 10 min in 1X PBS before application of blocking solution for 2 h at room temperature containing 1 µg/ml DAPI (Merck) and the relevant secondary antibody (goat anti-Rabbit IgG (H + L) Alexa Fluor Plus 488, #A32731; or Alexa Fluor Plus 647, #A21244; both Thermo Fisher, 1:1000 dilution). Samples were washed again (3 × 10 min in 1X PBS). For antigen retrieved mouse explant tissue a further application of anti-GFP antibody conjugated to Alexa Fluor-488 (#A21311, Thermo Fisher, 1:200, 2 h, room temperature) was performed at this point before final 3 × 10 min washes. Cell cultures were imaged on an EVOS FL in PBS buffer. Tissue sections were mounted under borosilicate coverslips as above and nail polish was used to seal the boundaries before imaging on a Lecia SP5-II inverted confocal microscope. DAPI nuclear fluorescence was excited with a 405 nm diode laser and captured between 415 and 481 nm on a PMT detector. EGFP or Alexa Fluor-488 was excited using an Argon-488 nm laser and captured between 498 and 577 nm on a HyD GaAsP detector in standard mode. Alexa Fluor-647 was excited with a 633 nm laser line and captured between 657 and 750 nm using a further GaAsP detector. Images were captured with 4 scan line averaging.

For quantification of AQP1 immunocytochemistry DAPI fluorescence highlighted nuclei while anti-Rabbit secondary Alexa Fluor 647 was used to label AQP1-primary antibody complexes. Confocal laser power was adjusted to remain within the dynamic range (0-255 grey values) relative to the brightest sample and maintained throughout. The same optical zoom was applied across all images taken through a 10X dry objective NA = 0.4 for lateral ventricle and third ventricle ChP. Fourth ventricle ChP did not occupy a single focal plane so Z-stacks were taken at 2 μm step sizes and maximum intensity was projected through the stack for each pixel to obtain an image for analysis.

Analysis of the confocal images was performed in Fiji/Image J [54]. For the lateral and third ventricles ChP was manually demarked by drawing around its boarder in one continuous line on the DAPI channel, then superimposing the same region of interest (ROI) onto the AQP1 channel and measuring mean intensity. For the fourth ventricle ChP was too complex to draw around manually. A manually drawn region inside the ventricle was specified, ChP within this region thresholded based on pixel intensity (DAPI channel), holes filled, then the ROI dilated to allow a narrow boarder. Gaps between adjacent bits of ChP were removed from the resulting ROI with the wand tool before projecting the finished ROI onto the AQP1 channel and measuring the mean intensity value. Slide background mean intensity values were also measured and subtracted from the initial results, though this did not vary much across sections or slides. ChP from 3 to 6 sections per region was assessed for AQP1 signal and the mean calculated.

### SURVEYOR assay

Cultured cells treated with AAV were washed in a 1:1 mixture of normal growth media and serum-free media as described above without AAV, trypsinised with 0.05% trypsin-EDTA (Thermo Fisher), resuspended in 1:1 medium and recovered by pelleting under centrifugation at 2348 xg, 5 min at 4 °C. Tissue samples were snap frozen on dry ice after collection and stored at -80 °C before thawing on ice prior to gDNA extraction.

A DNeasy Blood & Tissue Kit (Qiagen, Dusseldorf, Germany) was used to extract gDNA from cell line pellets or ChP tissue largely according to the manufacturer’s instructions with the modification that gDNA was eluted in 50 µl buffer AE. SURVEYOR assay was performed on gDNA as previously described [45]. Briefly, the *Aqp1* gene region where indel formation was expected to occur was amplified by PCR (Q5 High Fidelity 2X Master Mix, M0492S, New England Biolabs) and the PCR product purified using a QIAquick PCR purification kit (Qiagen). PCR product was denatured and reannealed to allow mismatches to form, if present, before 20 min digestion at 37 °C with 10 units of T7 endonuclease I (NEB). Digestion was stopped by addition of 0.8 units of proteinase K (NEB) and further incubation (5 min, 37 °C) before a second PCR purification and quantification by analysis of fragments with a DNA 1000 TapeStation assay on an Agilent 2100 Bioanalyser (both Agilent Technologies, Stockport, UK). Percentage of indel formation was calculated from band integrated areas using the method of Ran [55].

### Supplemental information

Additional methodological details are provided in Additional File 1, Supplemental Materials and Methods, including details of primers and ventricular system reconstruction using ITK-SNAP [56].

### Statistical analysis

IBM SPSS Statistics version 29.0.1.0 and G*power (RRID: SCR_013726) were used to analyse data with the statistical analyses used described in the results text or figure legends.

## Results

### AAV serotyped with the ShH10^Y445F^ capsid targets choroid plexus epithelium in vitro and in vivo

**Fig. 1:**
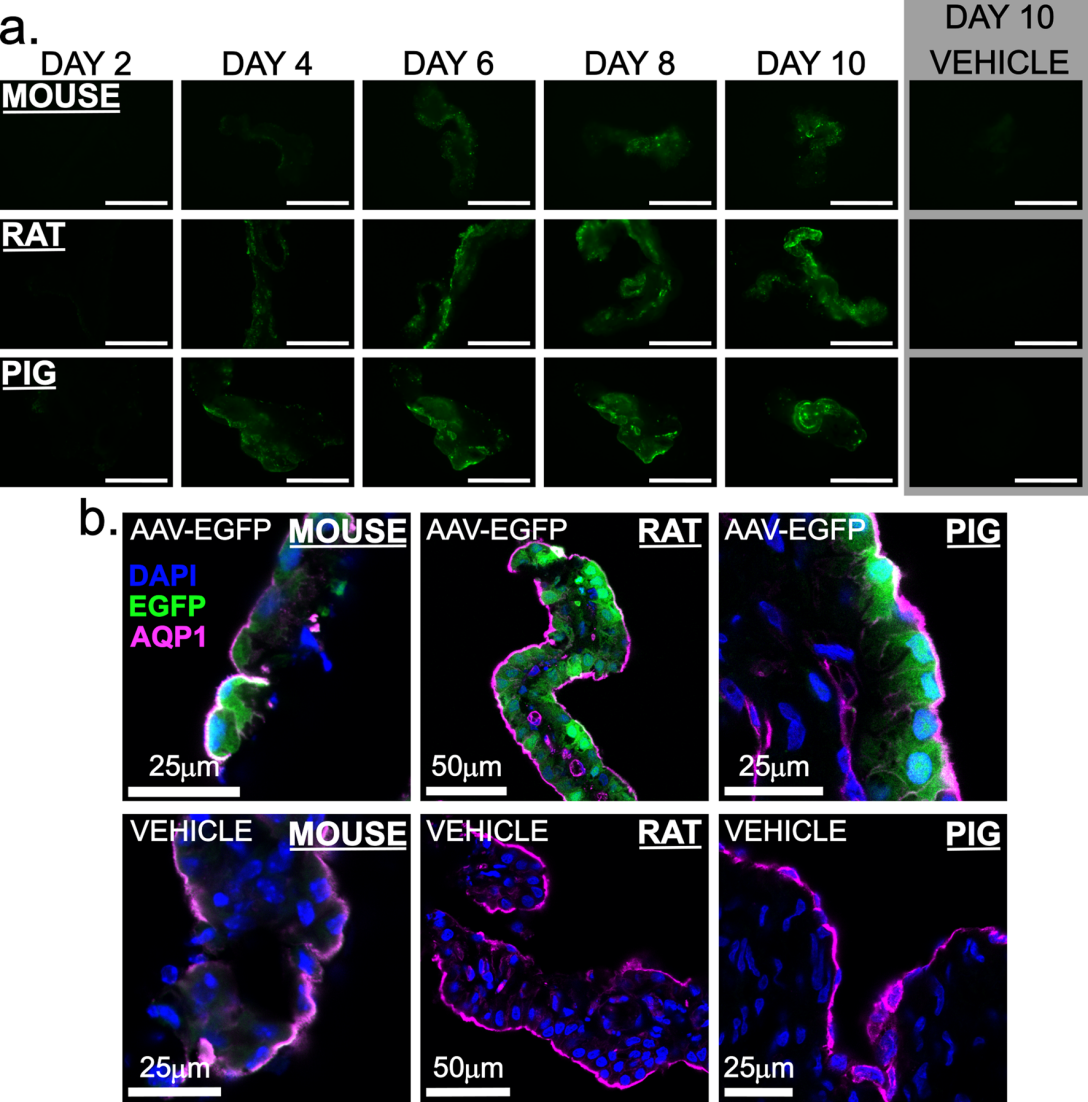
Targeting of choroid plexus epithelium by AAV transduction across species in vitro. **(a)** Choroid plexus was dissected immediately post-mortem from the species indicated and placed into explant culture medium in laminin coated wells. Vehicle (1X PBS) or AAV-ShH10^Y445F^-CMV-EGFP virus (3.189 × 10^10^ genome copies per ml media) was added to culture media at start of culture (day0). Explant cultures were photographed using an EVOS inverted microscope at the indicated times. Diffraction through the tissue in the DAPI channel indicates the presence of a tissue sample in the Vehicle control wells. Scale bars all 1 mm. **(b)** Confocal microscopy of 14 μm sections of fixed 10 day choroid plexus explant cultures showed EGFP expression (infection and transgene delivery) to choroid plexus epithelium with AQP1 staining on the apical membrane. No EGFP labelling is apparent if PBS vehicle instead of AAV was delivered

We began by testing the tropism of AAV-ShH10^Y445F^ to ChPE across species, circumventing high injection volumes in larger species by using in vitro preparations. Explant cultures of ChP from mouse, rat and pig demonstrated EGFP transgene transfer when co-cultured with AAV-ShH10^Y445F^ compared to vehicle only controls (Fig. 1a). Weak expression could be observed at day 2 in culture but became stronger by day 4 in all three species and was maintained to day 10.

To examine cell types in ChP explant tissue to which transgene was delivered by the ShH10^Y445F^ capsid, we undertook fluorescent immunocytochemistry on explant cultures after formaldehyde fixation at day 10. Sections of mouse, rat and pig explant tissue demonstrated clear EGFP expression in the outermost layer of the ChP cells with AQP1 co-expressed along the apical membrane (Fig. 1b).

**Fig. 2:**
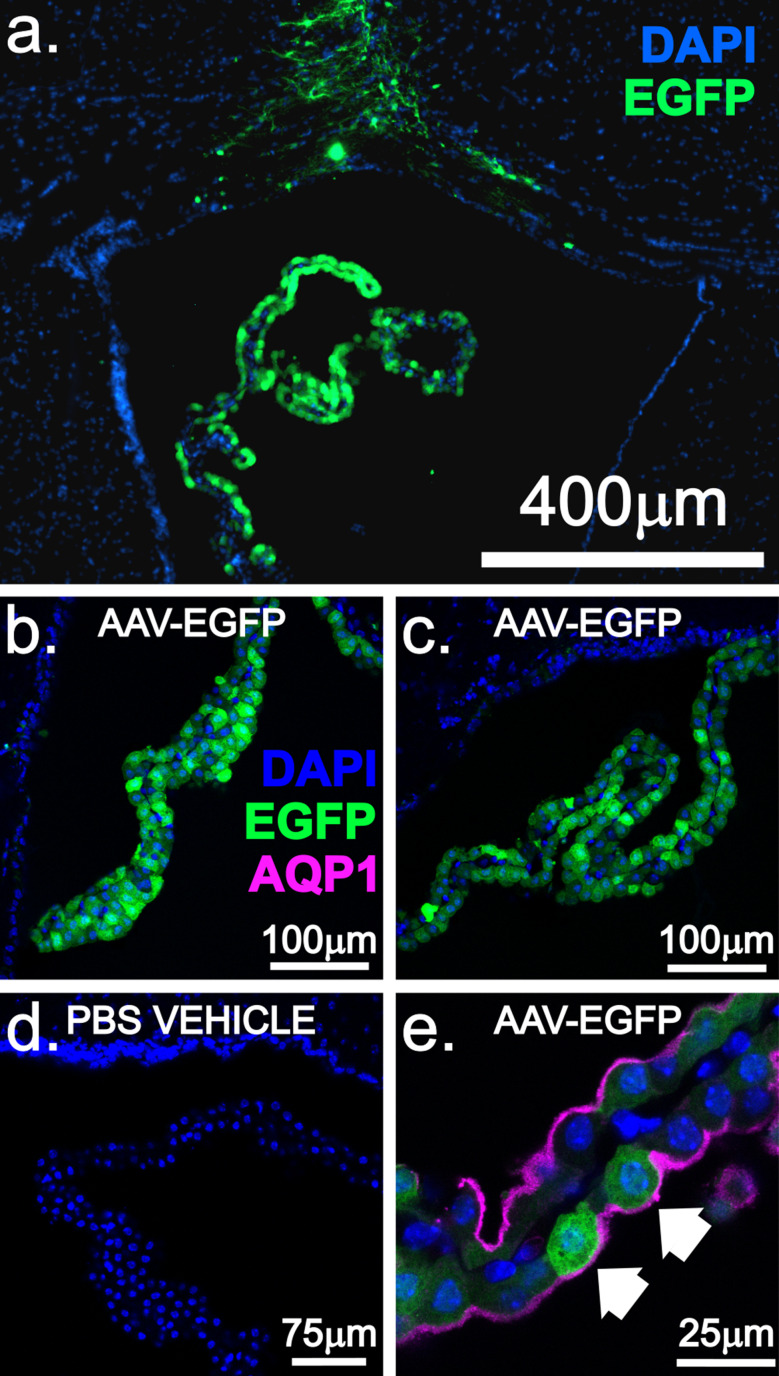
Mouse choroid plexus targeted by AAV transduction in vivo. **(a)** The choroid plexus and needle tract are labelled with EGFP following AAV transfer of this transgene by injection into the lateral ventricle. Few cells within the ependyma are labelled with EGFP. **(b**,** c)** Confocal microscopy resolves multiple individual cells within the choroid plexus labelled with EGFP following AAV transfer, which are not apparent when lateral ventricles are PBS injected **(d)**. **(e)** Confocal microscopy showing that EGFP labelled cells within the choroid plexus are on the surface and have AQP1 protein at the apical boarder (arrows)

We next tested the ability of ShH10^Y445F^ serotyped AAV to deliver a transgene to ChPE in the mouse brain by introducing 3.5 × 10^10^ genomes AAV unilaterally directly into the left lateral ventricle by stereotactic injection. Three weeks after injection histological analysis revealed EGFP expression within the ipsilateral ventricle ChP beneath the injection site (Fig. 2a). While the cells within the parenchyma following the needle tract also displayed EGFP expression, very few cells in the ependyma along the ventricle wall were observed to express EGFP. Individual cells at the surface of the ChP expressed EGFP which was not apparent after PBS vehicle injection (Figs. 2b-d). EGFP expressing cells were confirmed as ChPE cells due to the presence of AQP1 along the apical surface (Fig. 2e).

### Injected AAV distributes unevenly along the brain rostro-caudal and lateral axes

**Fig. 3:**
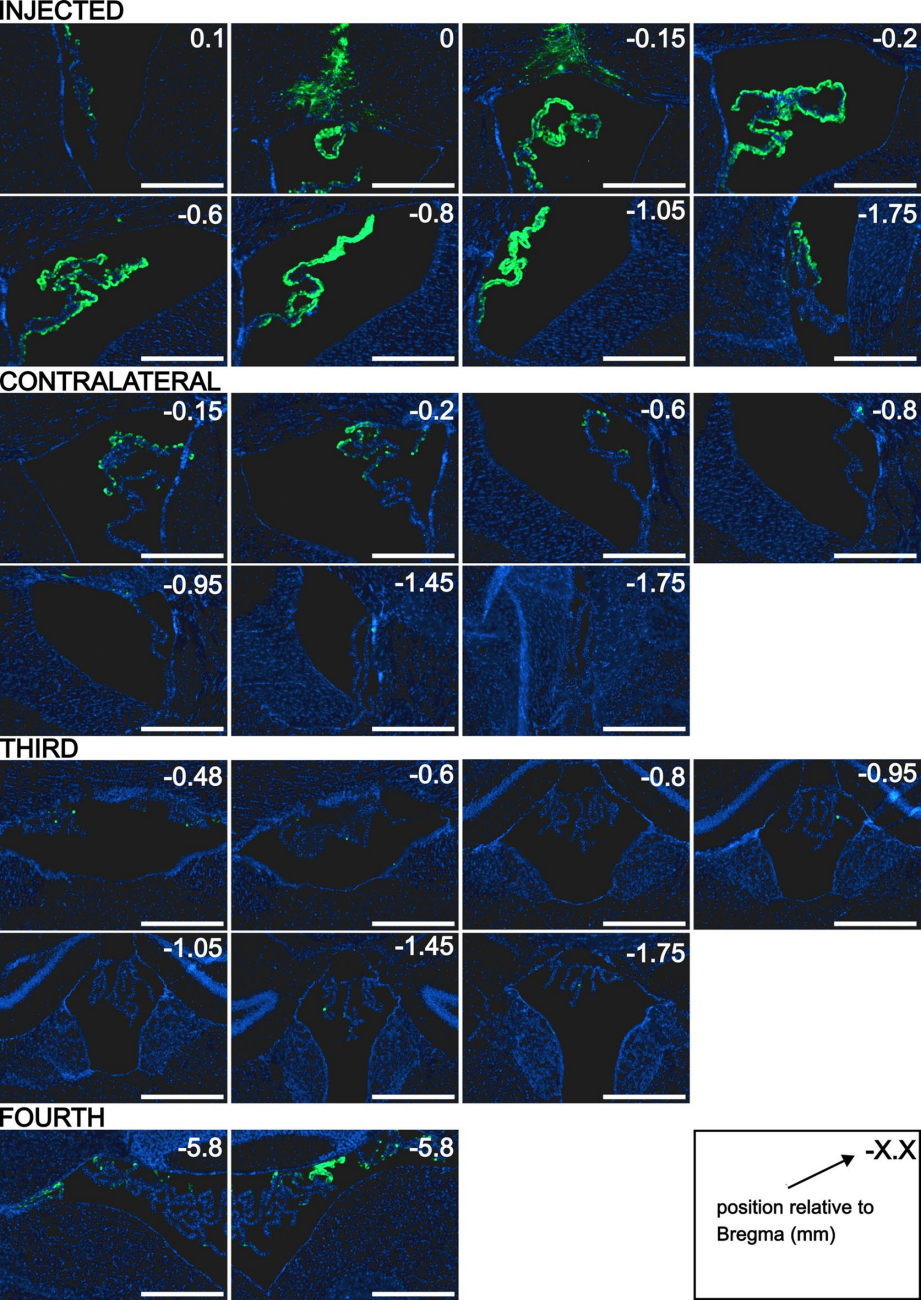
Distribution of AAV-mediated EGFP delivery to choroid plexus across the rostro-caudal axis of mouse brain. A total of 3.5 × 10^10^ genomes of AAV carrying the EGFP as a transgene was delivered in 10 µl volume into the left lateral ventricle at coordinates ML = -1, AP = 0, DV = -2. After 3 weeks mice were perfused and the localisation of EGFP observed by native fluorescence across the brain in histological sections counterstaining nuclei with DAPI, with particular focus on the choroid plexus in each of the major ventricular compartments. Scale bars all 400 μm

Transduction by AAV was also noted in the contralateral lateral ventricle and at lower levels in the third and fourth ventricles suggesting CSF movement could distribute AAV from a single unilateral injection across the ventricular system. To explore this further we systematically sectioned through the brains of mice injected with 3.5 × 10^10^ genomes of AAV to examine ChP located in all the ventricular compartments (lateral ventricles, third ventricle, fourth ventricle). Figure 3 shows a rostral to caudal representation of the location and intensity of the EGFP signal following unilateral injection on the left side. There was very little ChP rostral of the injection site but some of this did express EGFP. Ipsilaterally, AAV mediated-EGFP expression occurred in ChP directly at the site of ventricular injection but was strongest 0.8–1.0 mm caudally. At Bregma − 1.75 mm infection was considerably weaker and absent in a majority of ventral horn ChP. Infection on the contralateral side was considerably weaker but present mainly dorsally toward the ventricle roof and strongest between − 0.15 and − 0.6 mm relative to Bregma. Despite clear transit of AAV between the lateral ventricles through the foramen of Monro, third ventricle ChP was very poorly infected, though some green cells could be observed. AAV delivery of EGFP to the fourth ventricle ChP achieved comparatively more infection than the third ventricle. These results demonstrate that the expression of proteins within the ChP by intraventricular delivery of AAV is achievable.

### CRISPR/Cas9-mediated editing of the mouse Aqp1 gene in vitro

We next asked whether the ChP might be altered to reduce the production of a protein through an AAV delivery strategy. We re-examined a previously published CRISPR/Cas9 approach for knockdown of the apical water channel aquaporin-1 (AQP1), which displayed limited brain expression outside the choroid plexus epithelium (Fig. S1, Additional File 2). Staining specific to the presence of the anti-AQP1 antibody was confirmed (Fig. S2, Additional File 3).

**Fig. 4:**
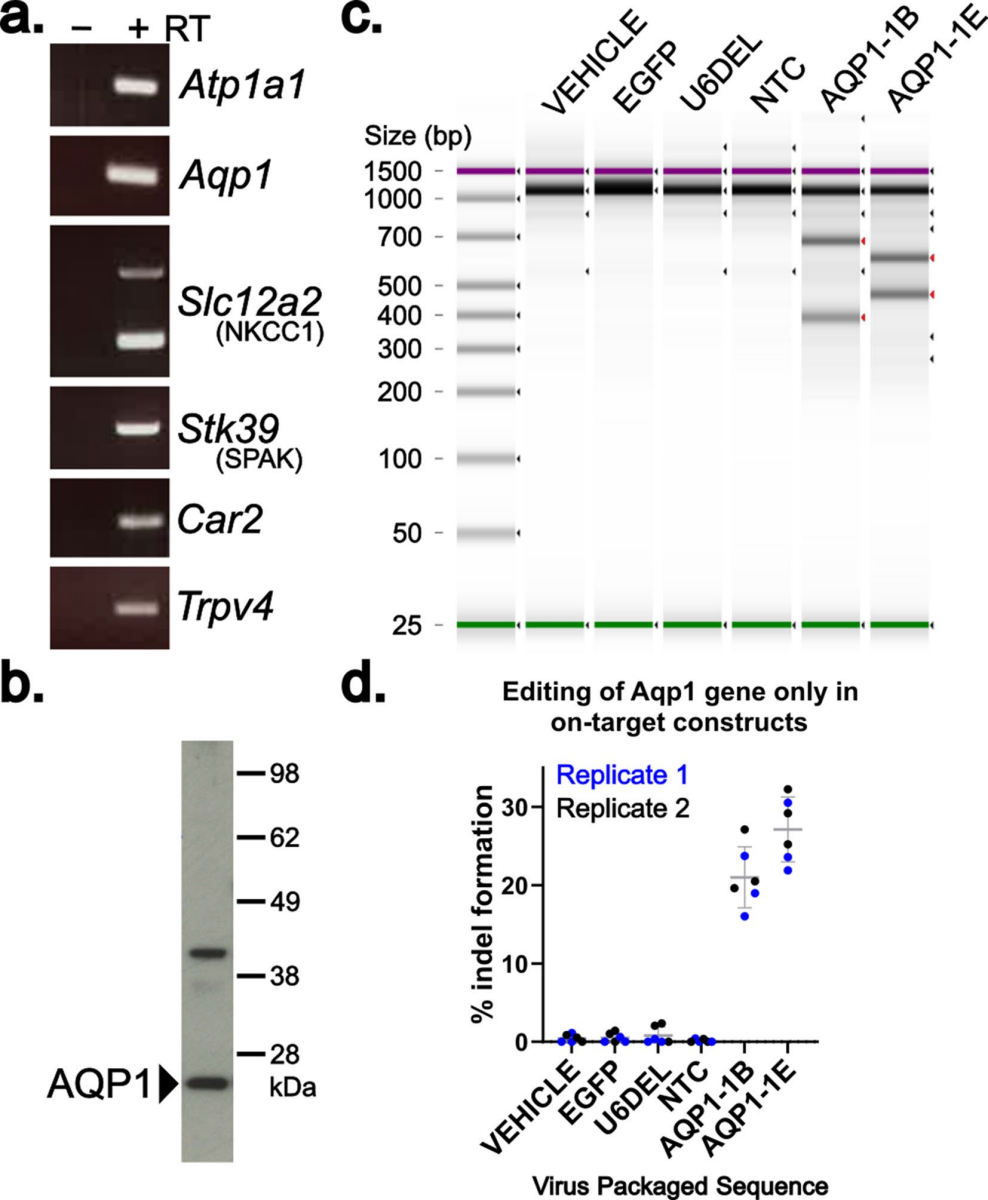
CRISPR/Cas9 mediated editing of the Aqp1 gene in a cell line expressing fluid production proteins. **(a)** Messenger RNA from B6-RPE07 cells reverse transcribed in the presence (+) and absence (-) of reverse transcriptase enzyme, then PCR amplified with primers targeting the indicated cDNAs. **(b)** Western blot with anti-AQP1 antibody showing AQP1 protein expression in B6-RPE07 cells, expected at 28 kDa. Additional bands may be post-translationally modified protein. **(c)** Representative DNA1000 TapeStation analysis for SURVEYOR assay showing indel formation at the targeted Aqp1 locus. **(d)** Quantitative analysis of indel formation, *N* = 6 from two independent replicates. EGFP, enhanced green fluorescent protein; U6DEL, U6 promoter and associated RNA scaffold and guide sequence deleted; NTC, non-targeting control

The mouse retinal pigmentated epithelial cell line B6-RPE07 was a useful surrogate for ChP in that it expressed multiple components of the fluid secretion pathway including Aqp1, the active subunit of the Na/K ATPase (Atp1a1), the Na-K-Cl cotransporter (NKCC1, Slc12a2), carbonic anhydrase 2 (Car2), SPAK kinase (Stk39), and transient receptor potential cation channel 4 (Trpv4) (Fig. 4a). B6-RPE07 cells clearly also expressed Aqp1 at the protein level revealed by western blot (Fig. 4b).

AAV driving the expression of SaCas9 and previously described targeting guide RNA sequences for mouse Aqp1 [45] could be shown by SURVEYOR assay to cut B6-RPE07 cell genomic (g)DNA at the Aqp1 target gene (Figs. 4c, d). Both guide RNAs (denoted 1B and 1E) allowed for SaCas9 cutting DNA at the expected position within the Aqp1 gene target. Negative control AAVs included EGFP (an alternative non-Cas9 protein), U6DEL (expresses SaCas9 but is absent the U6 promoter, guide and scaffolding RNA sequences), and NTC (non-targeting control containing a guide sequence not represented in human, mouse or rat genomes). Figures 4c, d shows none of the negative controls cut DNA within the Aqp1 sequence measured by SURVEYOR assay as expected, despite U6DEL and NTC expressing SaCas9 as for 1B and 1E targeting constructs (Fig. S3a, Additional File 4). The antibody used to detect SaCas9 through its C-terminal HA-tag in these constructs was validated as specific to the presence of the SaCas9 protein (Fig. S3b, Additional File 4).

### AAV delivery of targeted SaCas9 mediates Aqp1 gene editing in the choroid plexus epithelium

We next ICV injected AAV carrying guide sequences 1B and 1E at a 1:1 mixture (AAV-MIX-AQP1) previously successful in ocular gene therapy [45]. Due to the inefficient infection across the ventricle compartments in our EGFP experiment we used a higher dose of 1.0 × 10^11^ genomes delivered in the same 10 µl volume for targeted SaCas9 delivery.

**Fig. 5:**
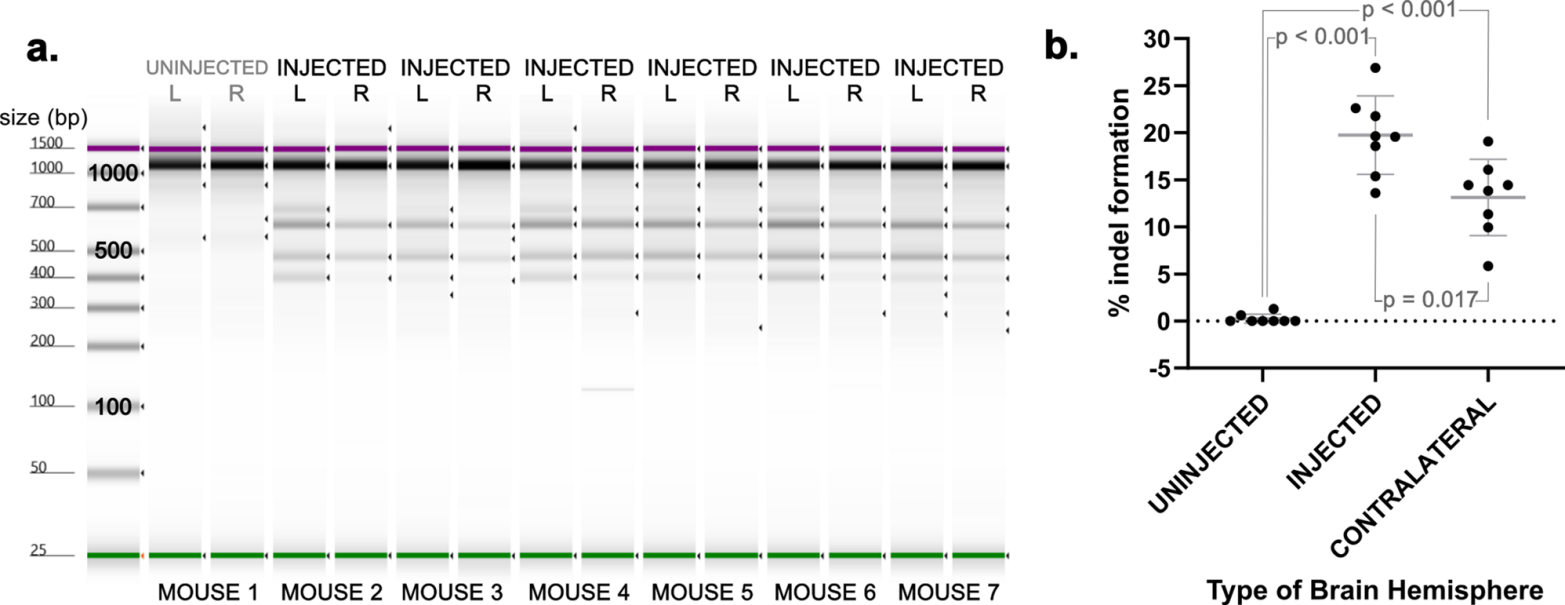
CRISPR/Cas9 mediated gene editing in both hemispheres following unilateral AAV injection. A higher dose of 1.0 × 10^11^ genomes AAV was injected compared with Fig. 3 to help overcome limited contralateral ventricle ChP transduction. **(a)** Representative examples of SURVEYOR assay DNA1000 TapeStation images used for quantification. **(b)** Percentage indel formation measured by SURVEYOR assay in choroid plexus obtained from individual hemispheres receiving treatment as described (injected with AAV, contralateral to AAV injected side, mouse receiving no injection). Mean ± S.D., *N* = 8. Percentage indel formation is significantly higher in injected or contralateral hemispheres compared to uninjected control hemispheres (Mann-Whitney U test). Percentage indel formation is significantly higher on the injected side compared to the contralateral hemisphere for the same mouse (Wilcoxon Signed-Rank Test, observed power 0.988)

Six weeks after injection, SURVEYOR assay showed indel formation at the *Aqp1* gene caused by AAV-MIX-AQP1. In gDNA isolations from ChP tissue extracted from the lateral ventricles, the formation of indels was apparent in ChP in both the injected and contralateral ventricles (both *p* < 0.001, Figs. 5a, b). Moreover, at this higher dose, although the percentage of indel formation on the contralateral side was significantly lower than on the injected side (*p* = 0.017, Fig. 5b), gene editing still occurred at two thirds of the level on the injected side.

### AAV delivery of targeted SaCas9 mediates Aqp1 protein knockdown in choroid plexus epithelium

**Fig. 6:**
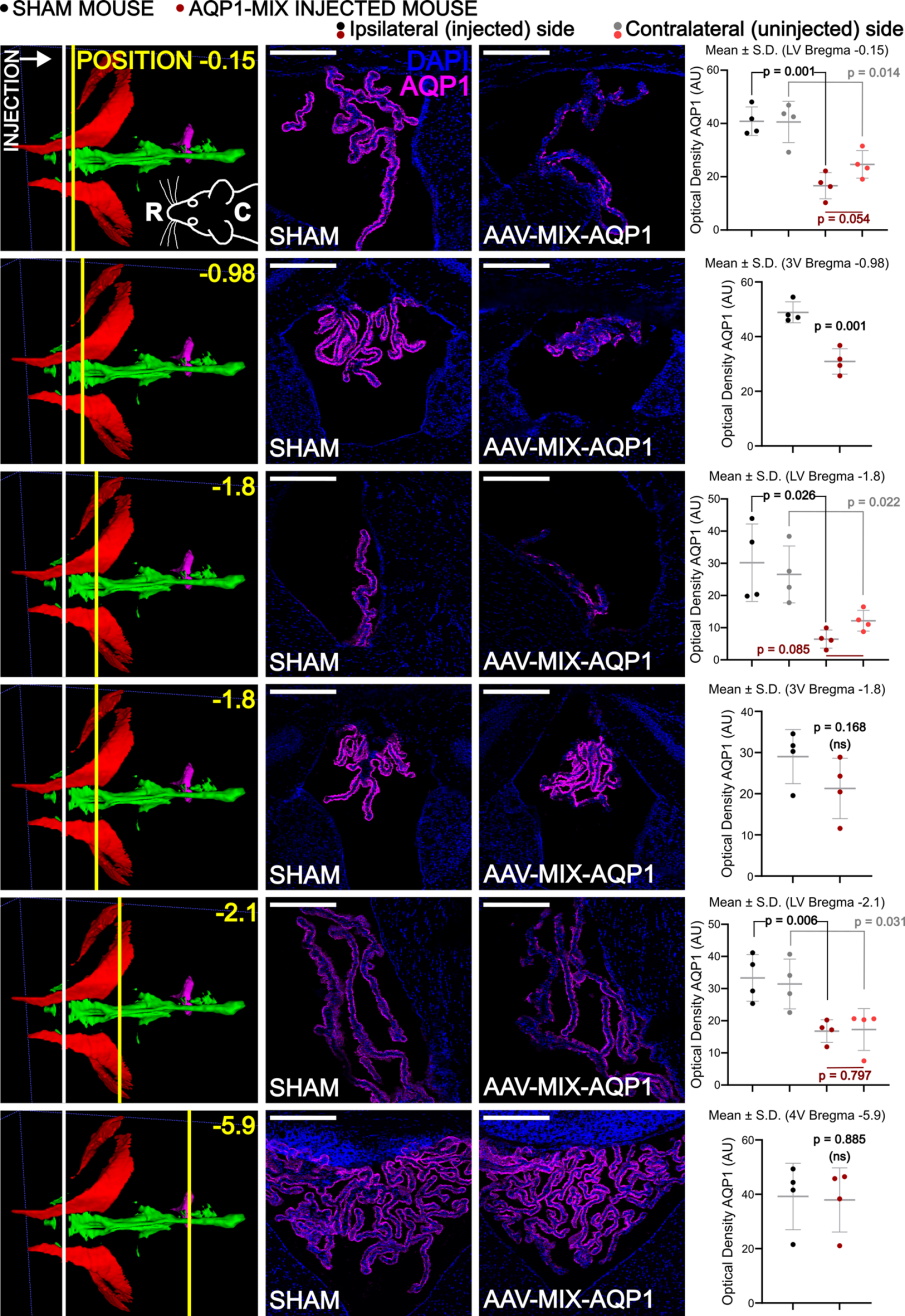
CRISPR/Cas9 mediated protein knockdown is spatially distributed across choroid plexus in different ventricles. Left panels show MRI scan 3D rendering of the mouse ventricular system (dorsal view; rostral (R) and caudal (C)), the approximate Bregma coordinates for the position of the sections examined across the row, and the injection site location at Bregma itself (white line). Middle panels show representative confocal images for the level considered in each row from SHAM injected mice (black/grey dots) or AAV-MIX-AQP1 injected mice (red/pink dots). A higher dose of 1.0 × 10^11^ genomes AAV was injected compared with Fig. 3 to help overcome limited contralateral ventricle ChP transduction. Scale bars are all 250 μm. Right panels show fluorescent immunocytochemistry for choroid plexus AQP1 protein optical density (AU, arbitrary units) at the rostro-caudal level considered across each row. Error bars are mean and standard deviation. Individual data points are calculated by averaging the AQP1 optical density from all sections measured per mouse at the level shown as displayed in Supplementary Figure S4, Additional File 5. Statistical analysis across groups (independent samples t-test, *N* = 4 per group) generated the p values indicated, taking *p* < 0.05 as significant. Observed power was 0.924-1.0 on the injected side in the lateral ventricles. Significant AQP1 protein knockdown is observed on both injected (darker coloured dots) and contralateral sides (lighter coloured dots) at all levels of the lateral ventricles (LV), and in the first part of the third ventricle (3 V) around Bregma − 0.98. Later in the third ventricle and in the fourth ventricle (4 V) AQP1 protein is not significantly reduced. Statistical analysis between injected and contralateral hemispheres of the same mice (paired samples t-test, *N* = 4 per group) yielded non-significant changes in AQP1 protein (*p* > 0.05), though power was lower than desirable (0.05–0.51)

Brains were sectioned at multiple levels through the rostro-caudal axis of the ventricular system and ChP analysed for AQP1 protein content by confocal imaging of fluorescent immunocytochemistry. Multiple sections were assessed per sample in each location (Fig. S4, Additional File 5) and averaged to produce a sample mean in each location assessed. AAV-MIX-AQP1 injection led to knockdown of AQP1 protein in the ChP six weeks post-injection (Fig. 6). Interestingly, the strongest knockdown was not observed at the injection site, but more caudally around − 1.8 mm relative to the injection site in the dorsal portion of the lateral ventricle. At this location 78% of protein is eliminated on the injected side and 54% on the contralateral side, compared to 59% of protein removed on the injected side and 39% on the contralateral side further forwards 0.15 mm behind the injection site. ChP in the ventral horn of the lateral ventricles (-2.1 relative to Bregma) was also infected with AAV-MIX-AQP1 achieving removal of 45–49% of AQP1 protein. Significant AQP1 knockdown was also evident in the first part of the third ventricle with around 37% of the protein removed at -0.98 relative to Bregma, but more caudally this effect weakened (26% protein removed at -1.8) and failed to reach significance (*p* = 0.168). At the level of the fourth ventricle AAV-mediated knockdown of AQP1 is insignificant (3%, *p* = 0.885). Comparison of lateral ventricle ChP AQP1 protein level between injected and contralateral hemispheres of the same mice revealed trends toward AQP1 protein being lower on the injected side at both Bregma − 0.15 (*p* = 0.054) and − 1.8 (*p* = 0.085) but not altered between hemispheres at Bregma − 2.1 (*p* = 0.797).

## Discussion

Although imperfect [29], using CSF flow for vector distribution has emerged as a favourable strategy [57]. AAV has become the most popular viral vector for brain gene therapy but specific ChPE targeting requires a refined infection profile. Proteins expressed from ChP-directed AAV might be novel therapeutic constructions, self or non-self, and could remain within the ChPE to confer a function not already attributed to the ChP, enhance an existing activity, or might be exported into CSF via the secretory pathway to influence the function of other CNS cell types. Gene therapies utilising this approach are expanding now spanning neurodegenerative conditions, psychoses, hydrocephalus and affective disorders [25–27, 58–60]. The principal of ChP/ependymal gene delivery has also been applied to metabolic disorders including those of lysosomal origin [29, 61].

In this study we present a novel variant of AAV2/6 that allows ChPE transduction with little concomitant ependymal infection. The AAV2/ShH10^Y445F^ variant infected ChPE across three different species in vitro. We further show the utility of this AAV vector to deliver a CRISPR/Cas9 system for the knockdown of AQP1 in the ChP by directed gene editing, mapping the spatial distribution of the editing effect across the ChP in all three ventricles.

In adult mice AAV2/5 and AAV2/8 display good infection of ChP but the wide variety of promoters, transgenes and titres used in these comparisons caution against overinterpretation [33]. As AAV2/8 also infects parenchymal cells AAV2/5, has become the serotype of choice for ChP targeting in both neonatal and adult mice [11, 27, 33, 58, 62], but failed to transduce either ChP or ependyma in cats after cisterna magna infusion [28]. We establish ChPE tropism of an engineered AAV serotype developed from AAV6. Using immunofluorescence and AQP1 counterstaining we show this serotype infects ChPE in mouse, rat and pig explant cultures (Fig. 1), and mouse ChPE in vivo after injection into the ventricle (Fig. 2). By confirming tropism across three mammalian species we present AAV2/ShH10^Y445F^ as a translatable capsid candidate that demonstrates strong transgene delivery to the ChPE and holds promise for gene therapy applications targeting this tissue. Appreciably, our investigation in vivo was limited to adult mice. How the AAV2/ShH10^Y445F^ serotype performs in neonates where AAV2/5 is established as the vector of choice and the ependyma is incomplete until the second week of postnatal life allowing leakage of AAV from the ventricle is unknown [63, 64]. A comparative study with matched transgene constructs in larger species of AAV2/5, AAV2/ShH10^Y445F^, AAV2/1 and AAV2/8 as the leading targeting serotypes is now also now warranted to progress gene therapy directed to the ChPE. AAV6 is capable of retrograde transduction beyond the injection site [30]. Although no evidence of such activity was observed in the present study a more comprehensive investigation of ShH10^Y445F^ in this regard may be appropriate.

AAV tropism depends on multiple factors that vary considerably across investigations including species and strain, serotype, dose, position at injection, delivery route and post-injection time frame [28, 29]. EGFP transgene expression delivered by AAV2/ShH10^Y445F^ was not observed in cortex nor parenchyma surrounding the ventricle spaces, while transduction of the ependyma was also poor (Figs. 2 and 3). While parent serotype AAV2/6 and 99% homologous AAV2/1 transduce neurons and glial cells across the brain in addition to ChP and ependyma [28, 38], we show in the mouse brain ICV injection of AAV2/ShH10^Y445F^ limited transgene expression to the ChPE aside from cells along the needle tract. Like other serotypes, it would be expected that AAV2/ShH10^Y445F^ can traverse the entire ventricular system, subarachnoid space and Virchow-Robin spaces and thus gain access to the parenchyma [65]. Transduction of cells in the needle tract shows AAV2/ShH10^Y445F^ has not lost tropism for parenchymal cells but may have been unable to penetrate tissue from either ventricle or Virchow-Robin spaces due to the altered tropism. Since direct injection of AAV5 into the striatum causes strong tissue expression [31], and AAV2/ShH10^Y445F^ infected cells in the needle tract, these serotypes retain transduction capacity for neural cells thus parenchymal exposure should be avoided. Refinement of the promoter used to drive the transgene would help to limit expression in undesirable locations and refine specificity to the ChPE [10, 66, 67].

Infection of ChPE without ependymal coinfection is challenging due to the similarity between these tissues [12]. ShH10^Y445F^ differs from AAV6 by four residues (I319V, Y445F, N451D, D532N), with three from ShH10 enabling epithelial targeting [43–46]. Y445F, introduced separately, enhances transduction by avoiding ubiquitination and proteasomal degradation, and may resist neutralising antibodies [48, 68, 69]. It also improves resistance to preexisting antibodies and tropism for airway, lung, and ciliary epithelium [47, 70]. It is probable that one or more of these modifications contributes to the relative selectivity for the ChPE over the ependyma. Combining Y445F with Y730F or F129L, known to double airway transduction [47], may further enhance epithelial targeting. AAVs utilise cell surface glycoproteins and co-receptors for attachment, internalisation and intracellular trafficking [57], whereby tropism is determined by the specific combination of proteins used. It is not known to which glycoproteins and co-receptors ShH10^Y445F^ binds to permit ChPE infection but the characterisation and mapping of these proteins in large animal models and patient populations would support translation.

Multiple studies have demonstrated experimentally induced protein overexpression within the ChP by viral-mediated transgene delivery. Conversely, downregulation of targets within the ChP is less well explored. RNAi approaches have enabled short-term protein knockdown [71, 72] and longer term shRNA-mediated suppression of adenosine A2A receptor activity to reduce pathology in a mouse model of multiple sclerosis [11]. We now report the application of CRISPR/Cas9 technology delivered via AAV to achieve negative regulation of a protein (AQP1) in the ChPE at the DNA level through non-homologous end joining (Figs. 5 and 6). As CRISPR/Cas9 generates a permanent frameshift in the protein genetic coding sequence, and ChPE cells renew very slowly [10, 12–14], we anticipate the effect would be durable persisting well beyond our six-week post-injection time point (Fig. 6), though follow-up work would be required to confirm this. Post-haemorrhagic hydrocephalus increases the expression of sodium-coupled bicarbonate exchanger NCBE (Slc4a10) and siRNA targeting this gene reduces CSF production and ventriculomegaly, while improving cognitive and motor performance in a rat model [73]. A shRNA or CRISPR/Cas9 adaptation of this approach would increase the longevity of the treatment toward clinical application. Application of CRISPR to novel therapeutics has been delayed by concerns about off-target mutation potential but with the conclusions of this article now under intense challenge current clinical trial data are awaited with optimism [74].

In most articles describing ICV delivery of AAV it is assumed the entire ChP is transduced. Here we fully describe the spatial distribution of AAV-ShH10^Y445F^ mediated ChP infection. In keeping with other reports [9, 17, 33, 60, 61], we show that a unilateral ICV injection of AAV-ShH10^Y445F^ was able to infect ChP in both ipsilateral and contralateral hemispheres (Figs. 3, 5 and 6). It is widely accepted this occurs independently of serotype as AAV1 [32], AAV9-PHP.B [75] and ShH10^Y445F^ (herein) exhibit the same patterns, produced by CSF cyclical movement generated via the cardiac cycle, respiration and the synchronous beating of ciliated ependyma [57, 76, 77]. Non-invasive imaging approaches have helped support these expectations and AAV delivered ICV to non-human primates spreads through the lateral ventricles of both hemispheres then throughout the CSF circulation [78]. Robust distribution to the lateral and third ventricles from lumbar or cisterna magna infusion was less convincing [77]. However, there may be dose dependency to the extent to which AAV can transit to the contralateral ventricle as contralateral infection with our lower dose of AAV (3.5 × 10^10^ gcs) was poorer compared to the higher dose (1.0 × 10^11^, compare Figs. 3 and 6). Poor contralateral infection from a single injection has also been observed by others with a similar low dose (2 × 10^10^ particles) [60]. While our high dose improved contralateral ventricle ChP transduction the distribution of AAV between hemispheres was uneven as gene editing was significantly lower on the contralateral side and protein knockdown trended toward lower efficiency contralaterally in two out of three areas examined (Figs. 5 and 6). Reduced penetration of the contralateral ventricle has also been reported for AAV9 [35], thus distribution of AAV into the contralateral ventricle via CSF dynamics is not efficient, but whether dose escalation could overcome the bias to the ipsilateral side without inducing toxicity has not yet been examined in detail. Alternatively, bilateral ICV injection demonstrates a more even distribution of AAV across the ventricular system with lateral ventricle transduction visibly similar on both sides [58].

Although the high dose (1.0 × 10^11^ genomes) in the AQP1 knockdown experiment improved AAV access to the third and fourth ventricles (Fig. 3 versus Fig. 6), it was clear that the majority of ChP at these locations remained uninfected in both experiments. A rostro-caudal gradient distribution for AAV2/5 has been reported by others [58], while AAV5 injected into the lumbar spine produced a reverse infection gradient with more infection in the fourth ventricle and less in the lateral ventricles [79]. Spatial distribution is better in neonatal mice with robust infection of ChP across both all ventricles [26, 80, 81].

Rostro-caudal distribution may be established by some combination of; (1) dose and dilution, (2) uptake of the AAV by the ChP, (3) anatomical and gene expression barriers to infection, (4) CSF flow and pressure in different compartments. Concentrated AAV is diluted in the CSF volume then enters ChPE cells in the rostral part of the ipsilateral ventricular system further diluting AAV. Thus, the rostro-caudal distribution observed could relate purely to dilution caused by AAV uptake. Inconsistent with this argument though, which predicts consistent dose-dependent gradients, we observed stronger infection around the margins of the fourth ventricle compared to the caudal parts of the third, stronger infection 1 mm behind the injection site compared to immediately beside it, and stronger infection in the ventral horns of the lateral ventricles further away from the injection site than within the closer third ventricle (Fig. 6). As suggested by Mazucanti [58], anatomical factors may contribute; the sheet-like structure of the lateral ventricles may permit broader AAV binding, whereas the convoluted morphology of the third and fourth ventricles may restrict AAV movement. Yet better infection of third and fourth ventricle ChP is achieved by AAV dosing caudally [79] indicating additional influences. Differences in CSF dynamics are also likely important. Fluid pressure and flow vary across ventricles [82]; the lateral ventricles have slower flow and higher pressure, while the narrower third ventricle has faster flow and lower pressure, with velocity decreasing again in the fourth ventricle. Faster flow in the third ventricle may limit AAV attachment and entry, explaining lower infection there compared to higher infection at the fourth ventricle margins (Figs. 3 and 6). Finally, differential gene expression in the ChP across the ventricular system may also influence AAV infection. Distinct profiles between the lateral, third, and fourth ventricles established embryonically [66, 83], along with changes driven by aging [84, 85], disease [86, 87] and circadian rhythms [88] could alter the expression of AAV attachment and entry receptors. Such variations may differentially effect serotype-specific infection and vary across developmental and physiological states [12]. It is a limitation of this study that tropism to ChP in different ventricular compartments was not examined in the explant culture system to determine if expression factors influenced AAV ChP targeting across ventricles. These factors likely contribute to complex models of AAV distribution in the CSF and may produce non-linear relationships with dose and ventricular volume during scaling to human brains. Distribution may also shift unpredictably in disease due to changes in CSF dynamics, gene expression, and ventricular structure.

The uneven AAV transduction profile across ChP throughout the ventricular system presents a challenge to application of gene therapy via this route and more work is required to overcome spatial biases we and others have observed for the effective delivery of CRISPR/Cas9 or RNAi targeting the ChPE. Various authors have considered the Trendelenburg position to aid distribution of other intrathecal drugs [89–91], while bilateral injection may also improve delivery to the third and fourth ventricles [58]. How the distribution described in mice herein relates to larger species is currently almost unknown, with little information about transduction patterns and AAV distribution across the ChP or ependyma in larger species [28]. Due to uneven spatial distribution gene therapy solutions resulting in ChP overexpression of a secreted protein for CSF distribution [25, 28] may succeed ahead of knockdown approaches as they are less dependent on near total ChPE infection. To our knowledge no studies have yet attempted to deliver secreted transgenes with AAV2/5 or ShH10^Y445F^.

## Conclusions

We characterised the tropism of the novel engineered AAV2/ShH10^Y445F^ demonstrating effective choroid plexus epithelium targeting across mouse, rats and pigs. This serotype confers effective and selective targeting of transgenes, including those with CRISPR/Cas9 gene editing function, to the ChPE following in vivo ICV delivery and facilitates work seeking to exploit the potential of AAV gene therapy. A single unilateral injection into one lateral ventricle unevenly distributes AAV with preferential infection of the lateral ventricles on the injected side over the contralateral, third and fourth ventricles. The development of reliable dosing strategies is required, integrating molecular and surgical approaches to overcome spatial distribution limitations and enable consistent access to the ChPE throughout the ventricular system. Hinderer’s statement that, “A single minimally invasive injection has the potential for radical transformation of therapeutic potential” [92] has not yet been realised, but some considerable progress is being made in this area and better awareness of translational requirements and further large animal studies addressing key translational questions would benefit the goal. AAV-mediated gene therapy for neurological conditions via manipulation of the ChP genome holds considerable long-term therapeutic potential but faces translational challenges.

## Supplementary Information

Below is the link to the electronic supplementary material.

Supplementary Material 1

Supplementary Material 2: AQP1 distribution in the brain.

Supplementary Material 3: AQP1 antibody testing.

Supplementary Material 4: Detection of and confirmation of HA-tag expression in cells transfected with plasmids containing SaCas9.

Supplementary Material 5: Individual section data averaged for Fig. 6.

## Data Availability

The datasets supporting the conclusions of this article are included within the article and its additional files. Raw image files analysed during the study are available from the corresponding author on reasonable request.

## Abbreviations

AAV: Adeno-associated virus
AOBS: Acousto-optical beam splitter
AQP1: (protein) or Aqp1 (gene) Aquaporin-1
ChP: Choroid plexus
ChPE: Choroid plexus epithelium
CMV: Cytomegalovirus
CNS: Central nervous system
CRISPR: Clustered regularly interspaced short palindromic repeats
CSF: Cerebrospinal fluid
DAPI: 4′,6-diamidino-2-phenylindole
EGF: Epidermal growth factor
EGFP: Enhanced green fluorescent protein
FCS: Fetal calf serum
gDNA: Genomic DNA
ICV: Intracerebroventricular
NA: Numerical aperture
NKCC1: Na-K-Cl cotransporter
NTC: Non-targeting control
PEG: Polyethylene glycol
PEI: Polyethylenimine
RNAi: RNA interference'
ROI: Region of interest
SaCas9: Cas9 nuclease from Staphylococcus aureus
shRNA: Short-hairpin RNA
siRNA: Small interfering RNA
U6DEL: U6 promoter and guide/scaffold RNA deleted
UCSC: University of California, Santa Cruz
