# Supplementary material for: Distribution of transgene in the rodent choroid plexus after intracerebroventricular injection of adeno-associated virus
Source: Fluids Barriers CNS. 2026 Jul 31;23:93. doi: 10.1186/s12987-026-00831-4 (PMC13428447; doi:10.1186/s12987-026-00831-4)
Supplement: Supplementary file 1 — Supplementary Material 1 [file 12987_2026_831_MOESM1_ESM.pdf]

Additional File 1.

Supplemental materials and methods

Cell culture

B6-RPE07 cells (gift from Dr Heping Xu, Queen's University, Belfast, UK) were grown at 37°C and 5% CO2 in DMEM (#D5671, Merck) supplemented with 5% heat-inactivated FCS, 2 mM L-glutamine, 1 mM sodium pyruvate, 100 units/ml penicillin, 100 µg/ml streptomycin (all Thermo Fisher). Cells were passaged every two days to a maximum of 20 passages and seeded to 24-well plates at a density of 60,000 per well or 6-well plates at a density of 400,000 per well. 24 hours later media was removed and replaced with a 1:1 mixture of normal medium and medium without FCS containing AAV at $1x10^9$ genomes/well or an equivalent volume of vehicle (1X PBS, pH 7.4). Cells were cultured for 72 hrs without further media replacement.

Western blot

Cultured cells or tissue were lysed in RIPA buffer (Merck) supplemented with protease inhibitors (Merck) and Halt phosphatase inhibitors (Thermo Fisher). Samples were incubated 30 min on ice after resuspending (cells) or grinding with a disposable pestle (tissue), then vigorously vortexed to complex lysis. Cleared supernatant was recovered by centrifugation (21,130 xg, 10 min, 4°C) before total protein quantified by BCA assay (Thermo Fisher). Aliquots of lysate were stored at -80°C until 5 µg (tissue) or 30 µg (cells) was mixed with NuPAGE LDS Sample Loading Buffer (Thermo Fisher) and dithiothreitol at a final concentration of 0.1M (Merck) for heat denaturation (95°C, 5 min) and loading onto NuPAGE 10% Bis-Tris gels run with Novex MOPS running buffer. Separated proteins were transferred to PVDF membrane using the iBlot2 system (all Thermo Fisher) and trimmed before blocking with 5% skimmed milk in Tris-Buffered Saline+Tween-20 (TBST) for 30 min at room temperature. Rabbit anti-AQP1 (ab168387, Abcam) at 1:1000 dilution in 5% skimmed milk

was incubated with membranes with gentle agitation at 4°C overnight. Rabbit anti-beta actin (#4970S, Cell Signaling Technology, Leiden, The Netherlands) was additionally used at 1:5000 dilution as a loading control. TBST was used to wash membranes 3x 10 min before secondary antibody incubation (anti-rabbit IgG HRP-linked secondary antibody, #7074, Cell Signaling Technology, 1:2000 in 5% skimmed milk, 2 hrs at room temperature). After further washing signal was detected using Amersham ECL Western Blotting Detection Reagents and Amersham Hyperfilm ECL (both VWR).

RNA extraction and RT-PCR

Total RNA was extracted from B6-RPE07 cells using a RNeasy mini kit together with on column DNase digestion (both Qiagen) following the manufacturer's instructions but eluting RNA into 50µl nuclease-free water. RNA was quantified by nanodrop and 2 µg was converted to cDNA using the iScript cDNA Synthesis Kit (Bio-Rad, Watford, UK) then amplified by PCR using Q5 High-Fidelity 2X Master Mix (New England Biolabs, Ipswich, UK) and primer pairs recognising the spliced mouse mRNA for *Atp1a1*, *Aqp1*, *Stk39*, *Car2*, *Trpv4* or both the spliced mRNA and un-spliced hnRNA of *Slc12a2*. Post-PCR, samples were run on a 2% agarose gel to resolve bands against a DNA ladder.

Primers for RT-PCR of mRNAs for Mouse Proteins Involved in Fluid Transport.

**Gene**	**Protein**	**Forward Primer Sequence (5'→3')**	**Reverse Primer Sequence (5'→3')**
Aqp1	Aquaporin 1	ACCTCCTCCCTAGTCGACAA	GCCAATGATCTCAATGCCCA
Atp1a1	Na/K ATPase Catalytic Subunit Alpha 1	TAGTCTCCAGCAACAGGACC	TGTCACCATGCTCCGATACA
Slc12a2	NKCC1 Transporter	ACCAACACCTACTACCTGCG	CATTCGCAAAGCCATCCTCA
Stk39	SPAK Kinase	CTGTGCACGTCCAGCTTTC	GCTTTATGGCTACGCGTTCT
Car2	Carbonic Anhydrase II	GCGTCCAAGAGCATTGTCAA	CATCAGATGAGCCCCAGTGA
Trpv4	Transient Receptor Potential Cation Channel Subfamily V Member 4	AAGAAAGCGCCCATGGATTC	TGGGCCGATTGAAGACTTTG

Magnetic resonance imaging (MRI)

Trans-cardial perfusion of a mouse under deep anaesthesia was performed with the aid of a peristaltic pump set to deliver approximately 8.3 ml/min. Initially 10-15 ml 0.22 µm filtered PBS pH 7.3 supplemented with 20 U/ml sodium heparin (37°C) was perfused to clear blood from tissues. Next 50 ml 4% formaldehyde in equivalent PBS without heparin was delivered at room temperature to fix tissues, before removal of skin, muscle and the mandible to preserve the brain within the skull. The skull was post-fixed in remaining fixative for 24 hrs at room temperature then washed daily in 4°C PBS for 7 days before scanning. A T2*-weighted, 50 µm resolution, coronal, anisotropic MRI scan with voxel size 50 x 50 x 31.667 µm was obtained at the Preclinical Imaging Facility, Radiobiology Research Institute, Department of Oncology, University of Oxford using a 3D multi-echo gradient-echo pulse sequence and a 26 mm coil (Rapid Biomedical, Rimpar, Germany) on a Varian 9.4 T MR scanner with Direct Drive console. Ventricles were segmented and volume rendered in ITK-SNAP software.

In situ hybridisation

Data for aquaporin-1 was recovered from Allen Brain Atlas dataset 79534912 as described.
