# Supplementary material for: Distribution of transgene in the rodent choroid plexus after intracerebroventricular injection of adeno-associated virus
Source: Fluids Barriers CNS. 2026 Jul 31;23:93. doi: 10.1186/s12987-026-00831-4 (PMC13428447; doi:10.1186/s12987-026-00831-4)
Supplement: Supplementary file 2 — Supplementary Material 2: AQP1 distribution in the brain. [file 12987_2026_831_MOESM2_ESM.pdf]

Additional File 2.

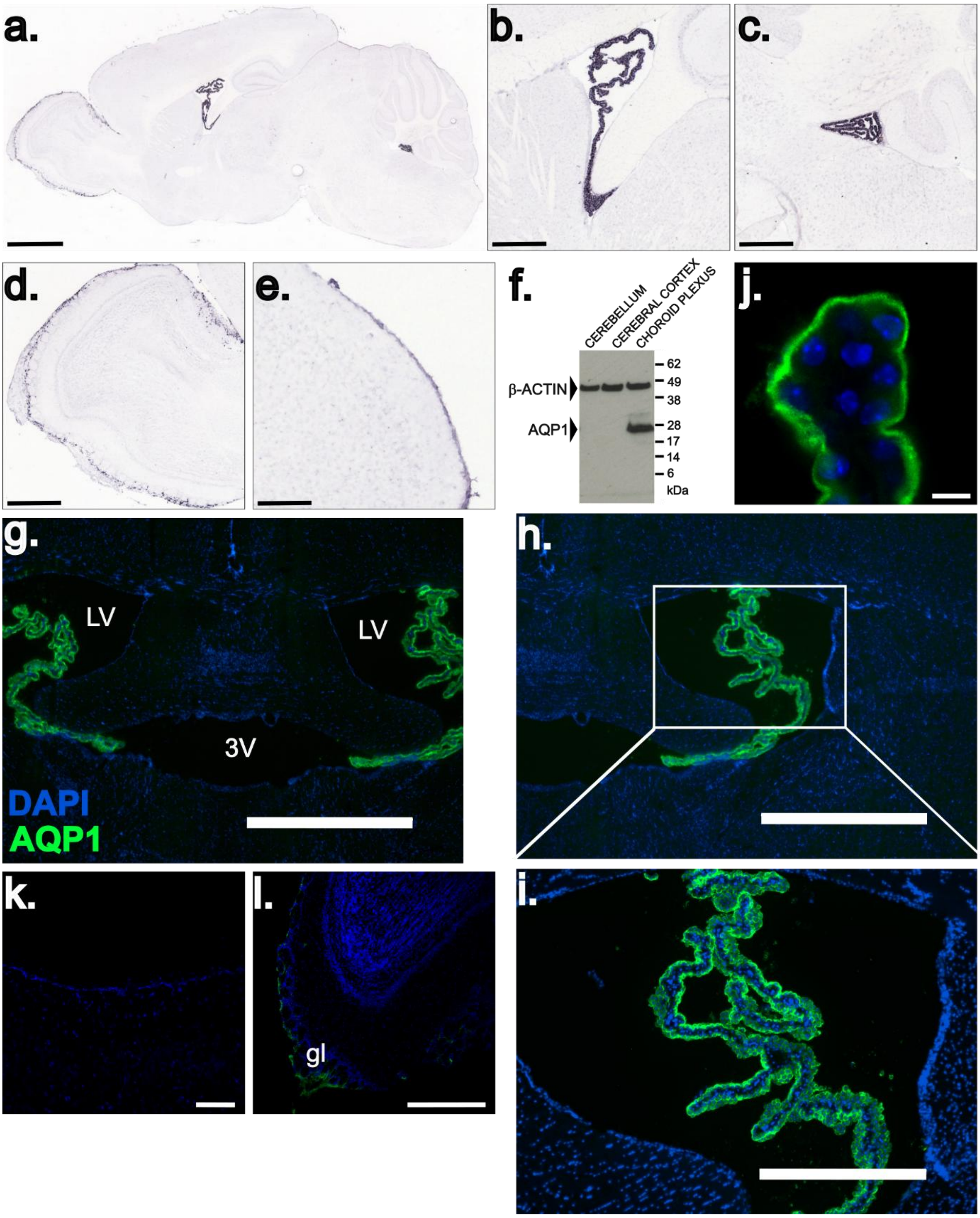

Supplemental Figure S1. Expression of Aqp1 in the adult mouse brain. (a-e) In situ hybridisation for Aqp1 mRNA in sagittal sections of the C57BL/6J mouse brain. Data using antisense probe RP_071018_01_A01 (Experiment 79534912) was recovered from the open-source Allen Brain Atlas [94, 95]. Image 12 showing restricted expression of Aqp1 across the

brain (**a**, scale bar 1678 μm), with zoomed segments indicating expression confined to the lateral and third ventricles (**b**, scale bar 420 μm) and fourth ventricle (**c**, scale bar 420 μm). In addition to isolated cells within the parenchyma that apparently expressed Aqp-1, expression was also evident within glomerular layer of the main olfactory bulb (**d**, scale bar 699 μm). Image 11 shows a zoomed segment of the brain surface, with pia matter labelling with the anti-Aqp1 probe (**e**, scale bar 175 μm). **(f)** Western blot indicating strong expression of Aqp1 protein within the choroid plexus but absent signal from the cerebellum and cerebral cortex. Aqp1 expected at 28 kDa and beta-actin expected at 42 kDa. **(g-l)** Fluorescent immunocytochemistry using anti-Aqp1 antibody and an AlexaFluor-488 labelled secondary antibody. Aqp1 protein is strongly expressed in the choroid plexus (**g-h**, scale bars 1 mm), with labelling of the apical surface of the choroid plexus epithelium apparent (**i-j**, scale bars 400 μm and 10 μm respectively). Almost no Aqp1 staining in the parenchyma or at the brain surface (**k**, scale bar 100 μm). Moderate Aqp1 staining of the olfactory bulb glomerular layer (**l**, scale bar 500 μm). Although Aqp1 mRNA expression was not absolutely unique to the ChP, at the protein level only the olfactory bulb contained AQP1 detectable by immunocytochemistry. LV = lateral ventricle, 3V = third ventricle; gl, glomerular layer.
