# Supplementary material for: Distribution of transgene in the rodent choroid plexus after intracerebroventricular injection of adeno-associated virus
Source: Fluids Barriers CNS. 2026 Jul 31;23:93. doi: 10.1186/s12987-026-00831-4 (PMC13428447; doi:10.1186/s12987-026-00831-4)
Supplement: Supplementary file 3 — Supplementary Material 3: AQP1 antibody testing. [file 12987_2026_831_MOESM3_ESM.pdf]

Additional File 3

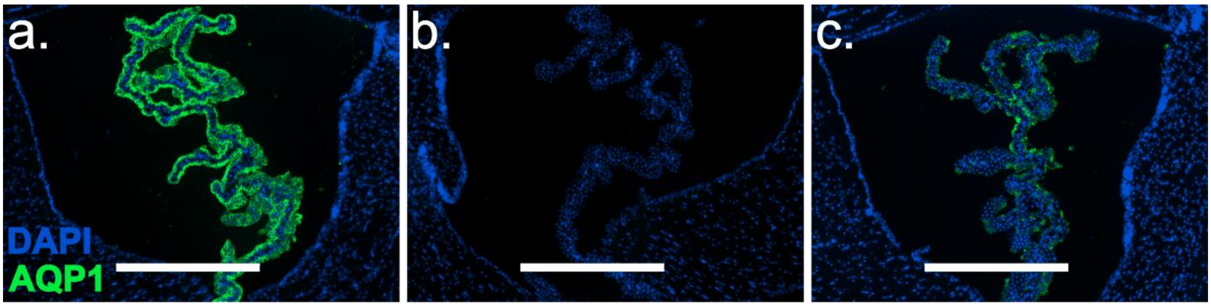

Anti-AQP1 + AF488 2° | Control IgG + AF488 2° | Anti-AQP1 + AF488 2° (no permeabilisation)

Supplemental Figure S2. Validation of AQP1 Fluorescent Immunocytochemistry Antibody. (a) Conventional staining with anti-AQP1 antibody and signal intensity around the apical membrane of the choroid plexus epithelium where AQP1 is expected to be located. **(b)** Staining is absent when the primary antibody is omitted and replaced with a non-immune IgG. **(c)** The antibody used was known to be directed against an intracellular portion of AQP1. In the absence of cell permeabilisation by omission of Triton X-100 from solutions, staining was considerably reduced as expected. Residual staining is due to the sectioning plane which may cut through or damage some cells. Scale bars are 400 µm. AF488 2°, indicates the presence of the AlexaFluor-488 conjugated secondary antibody.
