# Supplementary material for: Distribution of transgene in the rodent choroid plexus after intracerebroventricular injection of adeno-associated virus
Source: Fluids Barriers CNS. 2026 Jul 31;23:93. doi: 10.1186/s12987-026-00831-4 (PMC13428447; doi:10.1186/s12987-026-00831-4)
Supplement: Supplementary file 4 — Supplementary Material 4: Detection of and confirmation of HA-tag expression in cells transfected with plasmids containing SaCas9. [file 12987_2026_831_MOESM4_ESM.pdf]

Additional File 4

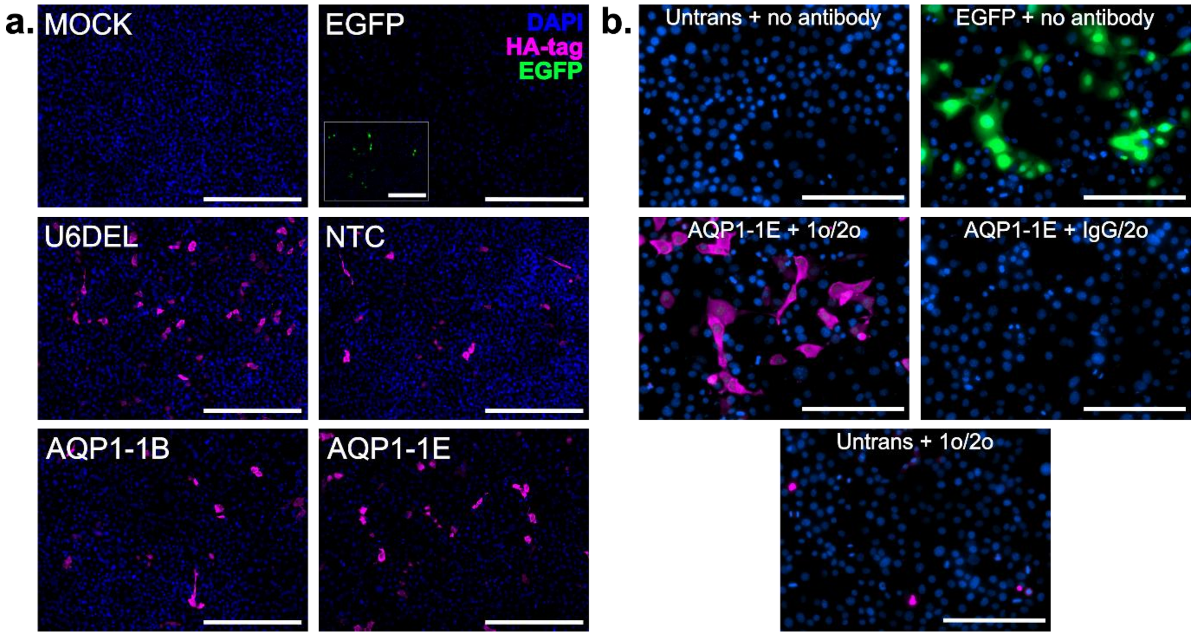

Supplemental Figure S3. Fluorescent immunocytochemistry of B6-RPE07 cells transiently transfected with the constructs indicated or MOCK (no DNA). (a) Negative control constructs U6DEL and NTC express SaCas9 (anti-HA tag staining) even though they fail to cut genomic DNA at the Aqp1 locus. Targeting guide sequences 1B and 1E result in delivery of the expressed SaCas9 to the Aqp1 locus for cutting of genomic DNA. SaCas9 was not observed as expected when a EGFP containing construct was transfected instead, with native EGFP fluorescence observable instead in the appropriate channel (inset). Scale bars all 400 µm. **(b)** Validation of the anti-HA primary antibody used to detect SaCas9 through a C-terminal HA tagging. Signal is detected only when primary antibody (1o), secondary antibody (2o) and cells transfected with SaCas9-expressing constructs (AQP1-1E) are present. No signal is observed is cells are untransfected (Untrans), transfected with an EGFP expressing construct instead (EGFP), or the primary antibody is replaced with a non-immune IgG (IgG). Scale bars all 200 µm.
