# Supplementary material for: Distribution of transgene in the rodent choroid plexus after intracerebroventricular injection of adeno-associated virus
Source: Fluids Barriers CNS. 2026 Jul 31;23:93. doi: 10.1186/s12987-026-00831-4 (PMC13428447; doi:10.1186/s12987-026-00831-4)
Supplement: Supplementary file 5 — Supplementary Material 5: Individual section data averaged for Fig. 6. [file 12987_2026_831_MOESM5_ESM.pdf]

# Additional File 5.

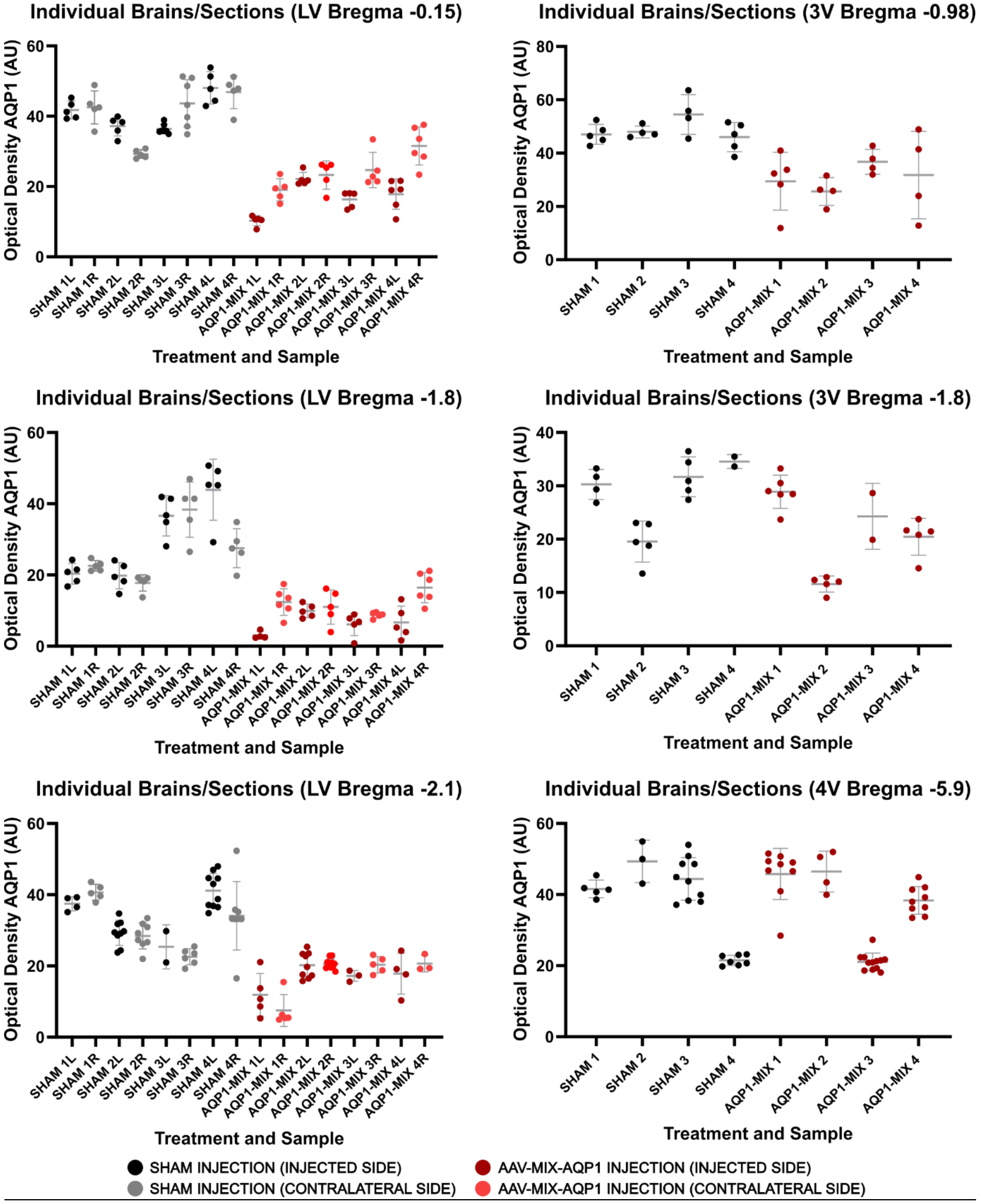


**Supplemental Figure S4. Optical density for AQP1 protein in Individual Sections.** For each coronal level examined across the rostro-caudal axis of the mouse ventricular system multiple sections were assessed for AQP1 protein levels by fluorescent immunocytochemistry giving the data points shown. The mean per mouse at each level (grey horizontal lines) was

carried forward to Fig. 6 for analysis across groups. Error bars show standard deviation for individual sections from each sample/mouse. Individual measurements show variability but an overall effect of AAV-MIX-AQP1 treatment.
